# Miscarriage communication in Australia: insights from women and general practice trainees

**DOI:** 10.1186/s12884-025-08004-1

**Published:** 2025-08-21

**Authors:** Joanne Wong, Jacqueline Frayne, Sarah Smith, Jade Bilardi, Meredith Temple-Smith

**Affiliations:** 1 Medical School, Department of General Practice, The University of Western Australia, Perth, Australia; 2 King Edward Memorial Hospital for Women, Perth, Australia; 3 School of Translational Medicine, Monash University, Melbourne, VIC Australia; 4 Medical School, Department of General Practice, The University of Melbourne, Melbourne, VIC Australia

**Keywords:** Miscarriage, Emotional care, General practice, Healthcare provider training

## Abstract

**Background:**

Miscarriage, defined as the unintended loss of pregnancy before 20 weeks affects 1 in 4 pregnancies in Australia. Despite its prevalence, the emotional impact is often overlooked, and can lead to many women experiencing psychological distress including depression, anxiety, and post-traumatic stress disorder (PTSD). Healthcare professionals play a vital role in providing support, however, many women experiencing miscarriage report dissatisfaction with their care, primarily due to the lack of emotional support. This study explores the perspectives of both women and General Practitioners-in-Training (GPiTs) to improve communication and provide actionable solutions for better miscarriage care during consultations.

**Methods:**

A qualitative study was undertaken. Recruitment occurred with consumers from pregnancy loss support networks, and GPiTs from the national General Practice training organisation in Australia. Online focus groups were conducted to discuss personal experiences, challenges and needs related to the miscarriage consultation. The data was transcribed, analysed using NVivo and coded using an inductive thematic approach. The research team identified key themes and reached a consensus on the findings.

**Results:**

Three key themes were developed:

*Emotional care*: Both women and GPiTs highlighted the need for improved emotional support, including acknowledgment of the loss, addressing guilt, and offering follow-up care. Women valued inclusivity and informed choices, while GPiTs reported challenges due to limited training.

*Provision of clear information*: Women preferred simple, clear explanations and written materials regarding miscarriage management. GPiTs recognised the importance of empathetic communication when conveying sensitive information.

*Training and skills*: GPiTs highlighted the need for early, formal training in miscarriage counselling whilst women emphasised the importance of education for all healthcare providers involved in miscarriage care.

**Conclusions:**

This study highlights the need to integrate emotional care into all miscarriage consultations. Additionally, early training to support and deliver a consistent approach in a sensitive manner, covering both the physical and emotional aspects of miscarriage is needed. This is essential to improve the quality of care provided, and ensure better support for women during this emotionally challenging time.

## Background

In Australia, miscarriage is defined as the unintended loss of a pregnancy prior to 20 weeks of gestation. It is a common complication of pregnancy, affecting around 1 in 4 pregnancies [1]. Despite its high prevalence, the experience of miscarriage is still often shrouded in silence, even amongst close friends and family [2]. While physically, miscarriage is managed relatively easily, the psychological impacts are often less effectively addressed [3]. Feelings of helplessness, fear, guilt, shame and grief following a miscarriage are common [4]. Psychological morbidity in women following miscarriage has been extensively studied over the past three decades, showing that significant psychological sequalae often results. Around half of women affected by miscarriage suffer from some form of psychological distress months to years after the loss [4, 5], with many meeting the clinical criteria for depression, anxiety and post-traumatic stress disorder (PTSD) [6, 7]. Furthermore, within one year of experiencing a miscarriage, women have a significantly higher suicide rate compared to women who have delivered a baby [8].

Although the experience of miscarriage is different for every woman, for many it can represent the loss of identity, of motherhood and a future child, and cause feelings of anxiety and doubt about the ability to procreate in the future [9]. The common occurrence of miscarriage and its relatively straightforward medical management may mean that health care professionals (HCPs) underestimate the psychological significance of an early pregnancy loss [9]. This is important, as the emotional support women receive at the time of miscarriage often shapes their experience and profiles their risk for development of adverse psychological outcomes [10]. Women who have lower levels of support from their partners and HCPs at the time of miscarriage tend to have stronger grief reactions and adverse psychological outcomes [10]. In contrast, women who are highly satisfied with the care provided by their HCPs tend to display better emotional adjustment [11]. Therefore, HCPs play a significant role in shaping the woman and her partner’s experience of miscarriage. Despite this, women often report high levels of dissatisfaction with the care they receive from HCPs during a miscarriage. Common concerns include a lack of acknowledgment of the woman and her partner’s loss, insensitive communication, the use of overly medicalised language, a focus on physical rather than emotional needs, inadequate information about miscarriage, and minimal support and follow-up services after the initial consultation [9, 12–14] As such, there is a significant mismatch between the care women seek, and the emotional support that HCPs believe they provide, or actually do provide. To address this discrepancy, this study forms part of a larger co-design research project aimed at creating a communication guide for HCPs, specifically General Practitioners-in-Training (GPiT) counselling women about miscarriage. While previous studies have explored the information women and their partners desire during a miscarriage, most research in this area has focused on hospital settings and from the perspective of nurses and midwives [15]. There has been little attention given to the perspectives of both women and HCPs, such as doctors, working in primary care [15]. This qualitative study aims to expand the existing body of knowledge on miscarriage communication by gaining deeper insights from both women and GPiTs, with the goal of offering actionable solutions to improve communication in these settings.

## Methods

### Research design

This research utilised a qualitative study design, aimed to further understand the needs of both consumers and HCPs in the miscarriage consultation. Consumers (women who have experienced miscarriage) and HCPs (in this case, general practice trainees - GPiTs) were recruited Australia wide to participate in focus groups.

### Ethics statement

Written, informed consent was obtained from all participants after they received detailed information about the study. This study was approved by the University Human Research Ethics Committee (reference number ET000039) and conducted in accordance with the Declaration of Helsinki.

### Recruitment

Given the qualitative nature of this study, a sample size calculation was not used as our aim was to not to quantify responses, but to capture diverse experiences and perspectives around the sensitive topic of miscarriage.

Consumers: defined here as women who had experienced miscarriage (up to 20 weeks gestation) > 3 months and < 2 years ago. This timeframe of > 3 months was chosen to allow participants time to process the experience but < 2 years to allow for more accurate recall of the event and the emotions associated with it. Consumers were recruited in June 2024 through known pregnancy loss support networks, such as Miscarriage Australia and Bears of Hope. A recruitment poster was distributed via both organisation’s social media channels.

Health Care Providers: defined here as prevocational general practice trainees (about to enter GP training) and GP registrars (trainees year 1 to 4), hereafter referred to as GPiTs. GPiTs were recruited in July 2024 from the Royal Australian College of General Practitioner’s (RACGP) training organisation, via email invitation and distribution of a recruitment poster. Purposeful sampling was used to include a diverse sample of GPiTs based on gender, state of residence, ethnicity and level of training experience. GPiTs, rather than fully qualified GPs, were intentionally selected for this study to gain insights into when they would have most benefited from miscarriage education. The research team believed that including fellowed GPs, whose training experiences vary depending on their graduation time, may have introduced too much variability into the data.

### Focus groups

#### Consumer focus group

Focus groups were held via Microsoft Teams and video recorded. Semi-structured interview questions were used to guide the discussion and explore how communication during miscarriage consultations could be improved. Focus groups were conducted as structured group interviews to ensure consistency across sessions. All were facilitated by the lead author (JW) and supported by the second author (JF), reducing variability and potential bias. Both are experienced general practitioners with strong communication and interviewing skills, which enabled them to probe effectively while fostering a safe and respectful environment for participants discussing this sensitive topic. Discussions involved participant’s personal experience about the miscarriage consultation, their experience with HCPs (all health providers involved in their care e.g. obstetrician, GP, nursing staff, midwives), how their experience could be improved and resources they would have found helpful for support. Field notes were taken for all focus groups by second author, JF.

#### GPiT focus group

Focus groups were held via Microsoft Teams and video-recorded. The same methodology, interview schedule, and facilitation approach were applied in the GPiT focus groups as in the consumer focus groups, with the lead author (JW) conducting all sessions and the second author (JF) providing support to ensure consistency and minimise bias. Semi-structured interview questions helped to guide the discussion, understanding what the GPiTs needs were in terms of providing miscarriage care to women, challenges they faced both in training and clinical practice, what information and resources they would find most useful and how they would ideally like miscarriage communication teaching to be conducted. Field notes were taken for all focus groups by second author, JF.

### Data analysis

The analysis of the focus groups followed a qualitative descriptive approach for thematic analysis. A qualitative descriptive approach was used to explore doctors’ and women’s experiences with miscarriage communication. The research team felt this method was appropriate for the sensitive nature of the topic and supported thematic analysis to generate participant-informed themes. The lead author, JW, was a final-year General Practice (GP) trainee at the time of the study. Following the focus groups, the video recordings were transcribed verbatim. JW re-watched the recordings and cross-checked the transcriptions for accuracy, a process which also facilitated data familiarisation. Field notes from both consumer and GPiT focus groups were reviewed as part of this process. An inductive approach to thematic analysis was employed, and the data was coded using NVivo version 15. Key themes were then identified, organised, and categorised. These themes, along with supporting quotes, were presented to the research team in a tabular format. Through rigorous discussion and reflection, the research team reached a consensus on the primary themes of the study.

## Results

### Participants

#### Consumers

Sixteen consumer advocates responded with expression of interest, with all invited to participate. Overall, 15 consumers confirmed attendance, with 3 unable to attend on the day due to personal reasons. All consumers were female, with a mean age of 30 (range 20–40 years). The number of miscarriages experienced by the participants ranged from 1 to 3. Some pregnancies resulted from natural conception, others from assisted reproductive technology. Two of the twelve women had experienced second trimester pregnancy losses. Participants lived in five of the eight states or territories in Australia.

Two 90-minute online consumer focus groups, each consisting of six participants were conducted by JW and JF.

#### GPiTs

Thirty-three GPiTs responded to the research advertisement. Overall, 18 GPiTs confirmed to attend, with two unable to attend on the day due to personal reasons. There were three males and 13 females distributed over the two focus groups, with a mean age of 34 (range 26–41 years). They represented all training levels in General Practice in Australia (pre-vocational, General Practice Training term 1–4). Six participants had completed additional training in gynaecology or were in the process of completing their advanced Diploma of the Royal Australian and New Zealand College of Obstetricians and Gynaecologist (DRANZCOG). Four participants had lived experience of miscarriage. There was a wide range of ethnicities represented in the GPiT group. Participants lived in six of the eight states or territories in Australia.

Two online 90-minute GP focus groups, each consisting of seven to nine participants were conducted by JW and JF.

Table 1 outlines consumers and HCPs characteristics.

**Table 1:** Participant demographics

Focus Groups	Consumer focus group Total *N* = 12		GPiT focus group Total *N* = 16
Age			
20–25	2		0
26–30	6		6
31–35	1		6
36–40	3		4
Gender			
Female	12		13
Male	0		3
Number of miscarriages		Training level	
1	6	Pre-GP training	1
> 1	6	GPT1	2
Gestational age at time of pregnancy loss		GPT2	7
First trimester	10	GPT3	5
Second trimester	2	GPT4	1
		Additional training (either as obstetric registrar or DRANZCOG)	6
		GPiT participants with lived experience of miscarriage	4
Ethnicity			
	Australian (11), Indian (1)		Australian, Peruvian, Chilean, Chinese, Argentinian, Egyptian, Iranian, Indian
Australian state in which participant resides			
WA	0		2
SA	0		1
QLD	2		1
VIC	3		3
NSW	5		8
TAS	1		0
ACT	1		1
NT	0		0

### Thematic analysis

#### Consumer and GPiT focus groups

**Fig. 1:**
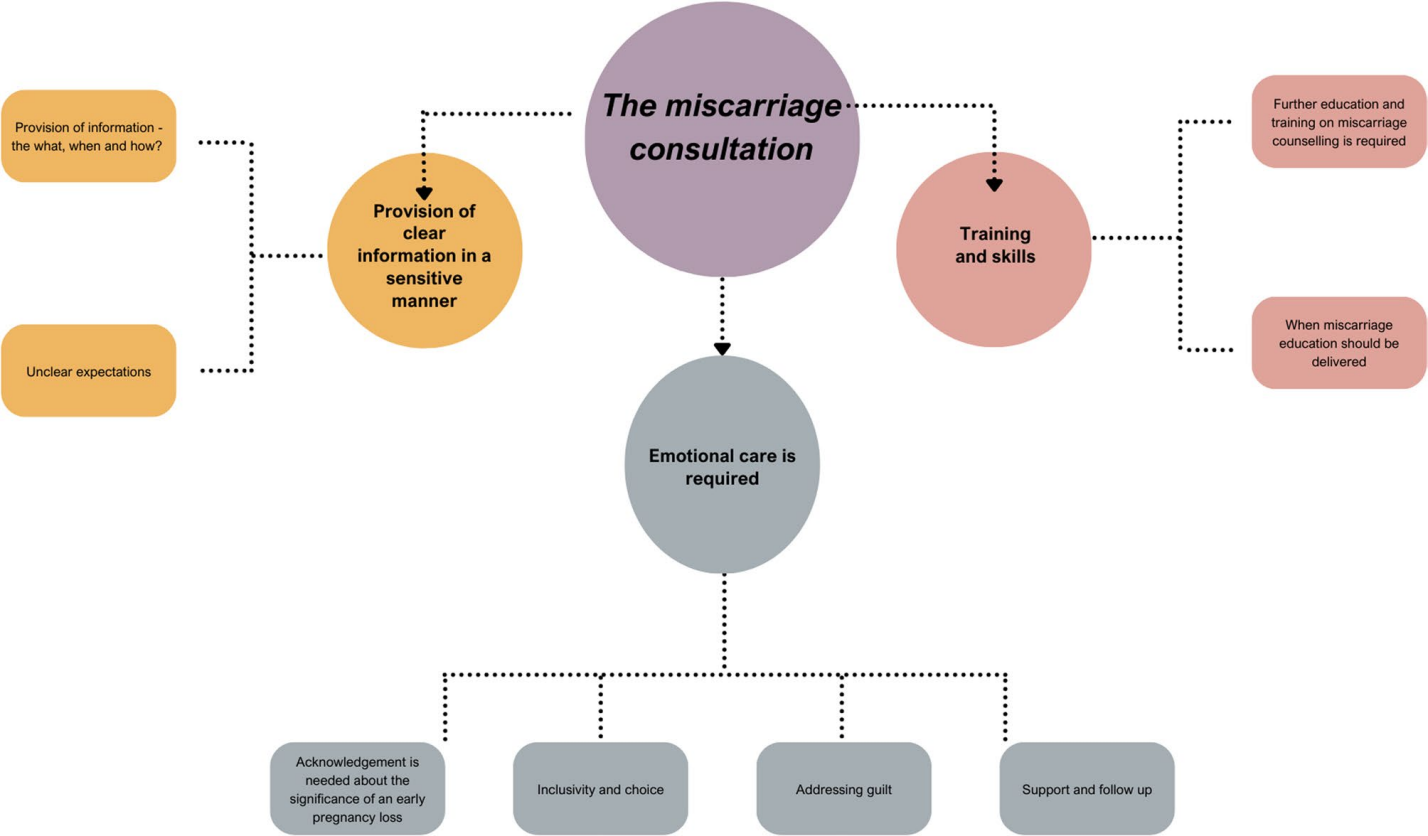
Thematic map

The thematic analysis of both consumer and GPiT focus groups resulted in three overarching themes, each with multiple subthemes (Fig. 1). The three main themes identified were: 1) emotional care is required, 2) provision of clear information in a sensitive manner, and 3) training and skills.

### Theme 1: emotional care is required

This theme highlights the importance of emotional care during early pregnancy loss. Both consumers and GPiTs agreed that acknowledging the emotional significance of the loss is essential, with expressions of sympathy being highly valued by those affected. GPiTs, however, often face challenges in providing such support, especially without sufficient training. Women highlighted the need for inclusivity and the ability to make informed choices during consultations. Addressing feelings of guilt and offering reassurance that the miscarriage was not their fault was another key aspect of emotional care. Follow-up support for both physical and emotional well-being was deemed important by both consumers and GPiTs, allowing for continuity of care and emotional validation following the loss. This theme emphasises the need for compassionate, individualised support to help women navigate both the physical and emotional complexities of miscarriage.

#### Subtheme 1a: acknowledgment of the significance of early pregnancy loss

To provide emotional care, both consumers and GPiTs emphasised that one of the most valuable actions a healthcare provider can take is to acknowledge the significance of a miscarriage. Prior to delivering any medical information, consumers valued an acknowledgment of their loss.

“It’s not the miscarriage itself… but the fact that he (the GP) just didn’t say sorry once. It just goes over and over in my head.”– *Consumer*.

“No matter how many weeks along they are, it’s important to just say I’m sorry about the loss of your baby…and that’s almost all you have to say in terms of an empathetic statement. It doesn’t matter how many weeks they were. It’s the loss of their baby.”– *GPiT*.

Despite the consensus among GPiTs that this acknowledgment was important, some felt they lacked sufficient training or experience to feel confident in offering such emotional support. GPiTs who had personally experienced early pregnancy loss noted that this experience led to a shift in their approach to consultations.

“My exposure to miscarriage was minimal. The advice from my supervisor at the time was limited to referring to the early pregnancy assessment service. I felt out of my depth during these consultations, but I wanted to provide better care… I just didn’t know how to do so within the time constraints and how to sensitively discuss options with patients.”– *GPiT*.

“Experiencing a pregnancy loss has changed the way I provide care… being on the patient side and then on the doctor side, you realise how much of an impact compassionate and empathetic care can make. I’ve slowed down during these consultations and focus more on the woman and her feelings.”– *GPiT*.

The focus groups revealed that acknowledging the loss and providing emotional care can be challenging for GPiTs. It is inevitable that some GPiTs will have personal experience with miscarriage. Therefore, participants highlighted that education about miscarriage should not only focus on the clinical aspects but also consider the emotional well-being of GPiTs. This would better prepare them mentally and emotionally for these sensitive consultations, helping them better understand the emotions they may encounter.

“I felt really uncomfortable when I was first starting to counsel patients about miscarriage and pregnancy loss. I think because it instils quite a lot of feelings of sadness even in yourself. And if you have your own experiences, it probably brings a little bit of that to the surface.– *GPiT*.

#### Subtheme 1b: inclusivity and choice

Women highly valued the opportunity for choice and inclusion in the miscarriage consultation. The language and terminology used during this process can be particularly emotive, and women expressed the desire to be asked what language they preferred during these sensitive discussions.

“A junior doctor came over to me and started talking to me about products of conception. I was using my baby’s name the whole time, and if I’m using my baby’s name, use my baby’s name. He had a name.”– *Consumer*.

“I know that I wanted to call it the baby initially, but then when I was going through the D + C [dilatation and curettage], it was pregnancy tissue… I didn’t want to hear that my baby was going to be removed. That’s a very confronting thing to think about.”– *Consumer*.

GPiTs acknowledged the importance of using empathetic and sensitive language.

“I think we are encouraged to do it (use empathetic language) in medical school… but sometimes we get lost in it. Once we’re in the silo of a specialty or general practice, we tend to use more scientific language.” - *GPiT*.

The focus groups highlighted that miscarriage care should be individualised, with management decisions guided by the woman’s values. Understanding a woman’s values was seen as an integral part of the consultation process. Some GPiTs, particularly those with an interest in birth trauma, recognised that women who are empowered to make informed decisions are likely to experience better overall health outcomes.

“I didn’t feel like I had a voice in the process. I just followed the protocol.”– *Consumer*.

“It’s not just about getting the woman to make the choice, but also feeling supported in their choice because, whatever happens, you definitely don’t want to push their hand or lead them into any particular option.”– *GPiT*.

“The level of trauma people experience is not solely related to the outcome of their birth but more to do with the experience they had and whether or not they felt listened to and validated.”– *GPiT*.

#### Subtheme 1c: addressing guilt

Both consumers and GPiTs agreed on the importance of addressing guilt during the miscarriage consultation. GPiTs emphasised the need to reassure women that the pregnancy loss was not their fault, as many women find comfort in this reassurance.

“I can see the doctor in my head saying it’s nothing you did or didn’t do. It just happened. That was really, really comforting in that initial emotional state.”– *Consumer*.

“These women are going through their head every single little thing they did… the time they ate that salad… the time they sat behind a smoker when they knew that they were pregnant… and then I go on to explain about the chromosomal abnormalities and that look of relief on their faces.”– *GPiT*.

#### Subtheme 1d: support and follow-up

Women expressed a strong desire for follow-up care and psychological support after a miscarriage. Many consumers preferred follow-up around the two-week mark to allow time to assess both their physical and emotional recovery. GPiT participants also recognised the importance of providing more holistic care by ensuring follow-up.

“We should encourage all patients to have follow-up within a couple of weeks… not just as a point of understanding what’s happened, but also as a point to follow up on their medical condition. I continued to bleed for six weeks but I wasn’t told that I should get help or be followed up.”– *Consumer*.

“I tend not to be in the headspace to be able to take on any information. I appreciated my health care provider sending me home and following up when I was ready to talk about what had happened.” — *Consumer*.

The GPiT focus groups also highlighted the need for supportive structures for emotional wellbeing for healthcare professionals involved in miscarriage consultations.

“Healthcare professionals also experience vicarious trauma. It’s important to acknowledge that miscarriage counselling can be emotionally triggering for a healthcare provider who may have experienced miscarriage themselves. There should be links to specific supports like the Doctor’s Health Advisory Service.”– *GPiT*.

“I guess as doctors, we probably prioritise barriers less and just have an awareness that it (counselling) can be triggering. Where do we seek help? Because often, we just shut down those feelings.”– *GPiT*.

### Theme 2: provision of clear information in a sensitive manner

Theme 2 highlights the need for clear and sensitive communication when providing information about miscarriage. Women expressed difficulty processing the often-large volume of verbal information. Women desired written materials to refer to later, especially regarding management options and potential complications. They also preferred simple, straightforward explanations, particularly for emotionally charged topics such as the causes of miscarriage and statistical prevalence. Many women did not want to discuss miscarriage statistics, particularly if they had experienced recurrent miscarriages. Clear, empathetic language about what to expect during and after a miscarriage including pain, bleeding, and emotional changes was highly valued. Women reported feeling unprepared for the physical and emotional aspects of the experience, and some GPiTs recognised that underestimating symptoms could lead to further trauma. Providing this information proactively could help reduce confusion, normalise emotional responses and prevent feelings of isolation, ultimately improving the overall care experience.

#### Subtheme 2a: provision of information– the what, when and how?

Women in our focus groups expressed difficulty in processing the volume of information typically delivered verbally by most healthcare providers during the consultation for miscarriage. Many women valued having written information to take home, particularly about processes such as management options and potential complications.

“I was furiously writing when my fertility doctor was talking to me about all the different options… not really listening.”– *Consumer*.

“When I saw all the blood… I had nothing to comfort me because I didn’t have any written information… I was relying on my memory at the time, and it was very emotional.”– C*onsumer*.

When it came to emotionally charged topics such as the potential causes of miscarriage, consumers expressed that they preferred very basic explanations.

“I was too emotional. I couldn’t take in anything else after the chromosome stuff. At the time, it was just give it to me simple and straight.”– *Consumer*.

“I think doctors are so desensitised to the language because it’s their everyday medical jargon… it hurts more hearing all these things as I just don’t understand what’s happening… I just need you to explain plain and simply for me to understand what is happening.”– *Consumer*.

Another emotionally sensitive topic was the discussion of statistical prevalence of miscarriage. Consumer focus groups revealed that women wanted choice about hearing these statistics. Most women were open to the discussion if delivered with emotional sensitivity. Those experiencing their first miscarriage found the discussion helpful, while those facing recurrent miscarriage preferred not to revisit statistics, feeling that often, the information provided by HCPs was not accurate for their personal situation.

“I’m getting told it’s one in four when I’m in my third miscarriage… and I don’t want to be told that anymore.”– *Consumer*.

Interestingly, GPiTs who had personal experience with miscarriage shared that whilst they had routinely discussed these statistics in clinical practice, they did not find them reassuring when they experienced a miscarriage themselves.

“I used to tell people statistics and think that would be reassuring. And then when I went through a miscarriage myself, I knew all the statistics and it didn’t matter. It was an emotional grief-focused event… and I just wanted people to say I’m sorry that happened to you.”– *GPiT*.

#### Subtheme 2b: unclear expectations about what to expect?

Women expressed a preference for direct, yet empathetic communication, with clear and realistic explanations about what to expect during and after a miscarriage. This included information on the expected amount of bleeding and pain, passing of pregnancy tissue, side effects of anaesthesia and/or medications, and the effectiveness of various management options. Many women in our focus groups described feeling unprepared for the pain and bleeding they experienced, particularly in cases of conservative and medical management. This led to confusion about whether their symptoms were normal or whether they required medical review.

“I got told it’s like a heavy period… I was horrified to see that much blood come out of me… and I’m like, is this normal? I don’t know, and I can’t go anywhere because there’s so much bleeding… I just didn’t expect it.”– *Consumer*.

“It’s not comparable to period pain at all and for my first miscarriage, the pain was very unexpected. For other friends and family who have had miscarriages, you just don’t get told that.”– *Consumer*.

“I have had two miscarriages and have had counselling from people (doctors) who, I guess just didn’t know that I had an idea of what was expected. And it’s really painful. Misoprostal is really painful and I’ve seen people come into hospital needing morphine…everyone has really different responses and reactions.” *– GPiT*.

Some GPiTs recognised that underestimating symptoms could result in further trauma.

“When we underplay pain, we also risk traumatising people because they’re not expecting it to be that bad.”– *GPiT*.

Additionally, providing information about potential emotional changes was considered essential. Women valued knowing that emotional reactions, such as grief, are a normal part of the process. Without this information, women may question whether their emotional state is “normal,” potentially leading to feelings of isolation or uncertainty, which could contribute to negative mental health outcomes.

“…even talking about the basics that you will experience grief and that’s completely normal… sometimes people don’t acknowledge what you’re feeling as grief because they don’t see it as a live thing, whereas it’s very much alive for you.” — *Consumer*.

“I struggled a lot with depression afterwards… I had suicidal thoughts… we have got to be told about what emotional changes we are going to be going through.” — *Consumer*.

“It’s later down the track that you see the trauma and complicated grief… we’re in a unique position to intervene… and presenting it to (GP) registrars that way, how to talk to patients when they’re going through miscarriage… there’s a far-reaching outcome.” — *GPiT*.

### Theme 3: training and skills

Theme 3 highlights the need for better training and education in miscarriage counselling for GPiTs. Doctors expressed a strong desire to improve their skills but noted the lack of formal training in this sensitive area, often relying on the guidance of more experienced colleagues. Consumers highlighted the importance of all HCPs, not just doctors, to receive training in miscarriage care to ensure compassionate and sensitive communication. The timing of this education was also a key concern, with many women experiencing inadequate support from junior staff who lacked an understanding of the emotional and medical challenges of miscarriage. Early and comprehensive training, particularly during medical rotations like those in emergency departments, was seen as essential to prepare all doctors with the necessary knowledge and skills to offer the best care to women experiencing pregnancy loss.

#### Subtheme 3a: further education and training on miscarriage counselling is required

A common theme amongst doctor participants was a strong desire to perform well in their role, but a significant lack of the necessary skills and training. None of the GPiTs in our study had received formal training in miscarriage counselling before supporting women through miscarriage. Instead, all relied heavily on the guidance of senior staff, with varying levels of educational support depending on the supervisor.

“My first experience of miscarriage was as an intern during my first term in the emergency department. I had to break bad news about miscarriage, about three to four times. This was more than I expected and I had no preparation for it at all.” — *GPiT*.

“Junior doctors are the most vulnerable medical professionals… they really want to do this well but don’t have the training… I think it’s really important to provide education as early as you can.” — *GPiT*.

“I think residents usually get the job of discussing miscarriage and are thrown into the deep end without really knowing what to say or how to say it in a compassionate manner.” — *GPiT*.

Similarly, consumers in our focus groups expressed the need for all HCPs (including doctors, nursing staff, midwives and allied health professionals) to receive training in miscarriage communication to improve their own experiences.

“I think it (miscarriage communication education) should be extended to different health professionals that support the process… because when I called the birth suite, they should have been the most sensitive to when a pregnant person is calling in relation to their anxieties about potential pregnancy loss.” — *Consumer*.

“It would be great to re-educate (miscarriage communication) all in medical fields because unfortunately there are some people who just don’t understand the sensitivity of this whole conversation.” — *Consumer*.

#### Subtheme 3b: when miscarriage education should be delivered

The timing of when miscarriage education should be provided was also a key topic in the focus groups. Consumers shared that they often found themselves being seen by junior staff who had limited understanding of the complexities they were facing.

“I’ve had more than one miscarriage, and when I went in for the third time, the junior doctor did not think to ask about my history before trying to placate me about how it’s quite common, which is a little insensitive.” — *Consumer*.

“I’ve known about it (miscarriage) and seen a lot of it myself around my workplace, so it (miscarriage) was not something out of the blue, but at the same time, having that experience myself was quite jarring, and I felt I knew more information than what some HCPs were giving me, which was quite inappropriate as this information should have been coming from my HCP whether they were a junior doctor or a specialist.” — *Consumer*.

GPiTs also discussed the variability in training and the importance of providing education early in their careers. Rotations specific to women’s health were often optional, and even when doctors chose to undertake additional training, many of their encounters occurred prior to this education.

“As an intern in the ED [emergency department], you get protected teaching. This would be a super helpful topic [miscarriage counselling] to be covered because for junior doctors who are doing an ED term, you may invariably come across these patients.” — *GPiT*.

“If this is taught during the ED rotation, you’re not only reaching GPs, but you’re reaching every doctor… because so much of miscarriage counselling happens in emergency, but many doctors won’t get this training.” — *GPiT*.

## Discussion

The findings from this study offer a comprehensive view of the experiences and perspectives of both consumers and GPs-in-training involved in miscarriage care in Australia. Thematic analysis highlighted three key areas: the necessity of emotional care, the importance of clear and sensitive information provision, and the need for further training and skill development in miscarriage counselling. These themes reflect the challenges surrounding miscarriage care and highlight the need for a more empathetic, individualised, and well-supported approach in clinical practice.

One of the most prominent themes emerging from both the consumer and HCP focus groups was the need for emotional care in miscarriage consultations. This finding aligns with previous research suggesting that miscarriage is not only a medical event but also a deeply emotional experience [15–17]. Both consumers and GPiTs agreed that acknowledging the loss and expressing empathy is essential. The significance of offering a simple, empathetic statement such as, “I’m sorry for your loss” was emphasised across both groups. Many women noted that the lack of such an acknowledgment left them feeling unheard and unsupported. This aligns strongly with previous research in the field [15].

For GPiTs, the delivery of emotional care can be personally challenging and many admitted feeling unprepared or uncomfortable in addressing the emotional aspects of miscarriage. Addressing the emotional needs of both consumers and GPiTs is vital for creating a supportive and effective healthcare environment [18]. Several participants felt strongly that GPiTs should be educated about the potential emotions that may surface and have access to psychological support, especially when the experience of miscarriage counselling may provoke reflection of their own experience or elicit certain emotions. This reciprocal emotional care is essential for the sustainability of compassionate practices and to prevent burnout in GPiTs [18]. In turn, it will likely have flow on effects, improving the quality of holistic care for patients.

The provision of clear, accessible, and empathetic information emerged as another key theme from the focus groups. Women expressed difficulty processing the often-overwhelming amount of information presented to them during the consultation. Many recalled being too emotionally distressed to understand complex medical details, such as, the potential causes of miscarriage or the specific details of options available for managing pregnancy loss. As a result, women in this study highly valued written information they could refer to at home. This finding highlights and aligns with previous studies regarding the importance of providing written resources to assist patients in processing the information [15].

Further, the delivery of statistical data on miscarriage was a point of contention. While some women, especially those experiencing their first miscarriage, found it reassuring to know that miscarriage is common, others with recurrent pregnancy losses found such statistics to be discouraging and unhelpful. This highlights the need for individualised approaches to information delivery, tailoring communication to the emotional and psychological needs of each woman [3]. This personalised approach may improve a woman’s understanding of the situation and reduce their emotional distress [3].

Women in this study expressed a need for clear and realistic expectations about the physical and emotional aspects of miscarriage. Participants reported feeling unprepared not only for the intensity of bleeding and pain, but also the short- and long-term emotional impact of miscarriage [19]. Inadequate communication around physical (pain and bleeding) and emotional symptoms (grief, guilt, depression) is a significant concern, as it can exacerbate the trauma associated with miscarriage and leave women feeling confused about their symptoms and isolated in their grief [15]. Both consumers and GPiTs in the study emphasised that being clear about both the physical and emotional symptoms would help women mentally prepare and reduce potential stress during the process. Women highlighted the importance of receiving this information in a direct yet compassionate manner. Setting expectations in advance would help women understand what to expect physically and emotionally, minimising confusion and fear during the process.

Another significant finding from this study was the widespread recognition that GPiTs lack sufficient training in providing compassionate and sensitive miscarriage counselling. None of the participants in the GPiT focus groups had received formal training on how to provide emotional care. Rather, they relied heavily on the guidance and experience of senior staff members, which often varied in quality and consistency. This variability in training was particularly evident amongst junior doctors, who expressed a strong desire for more structured and formal education on miscarriage care.

The lack of training in miscarriage counselling has broader implications for patient care. HCPs who feel unprepared or uncomfortable in delivering sensitive information may inadvertently contribute to the emotional distress of their patients [14]. Several GPiTs highlighted a gap between their medical knowledge and their ability to communicate effectively with women experiencing miscarriage. While emotionally sensitive counselling is briefly addressed under ‘Words of Wisdom’ in the Australian General Practice Training Curriculum, this suggests a potential need for more formal and structured education on the topic within medical training [20]. Furthermore, education should be made available to all healthcare professionals who may be involved in miscarriage care, including nurses, midwives, allied health workers, and emergency department staff.

The timing of this miscarriage training is also important. Many consumers in this study noted that they often encountered junior doctors with little understanding of the emotional and psychological challenges of miscarriage. A more integrated approach to education, where miscarriage counselling is taught early in a GPiT’s career and reinforced throughout their training, would likely lead to better patient outcomes. For Australian GPs-in-training, women’s health rotations are often optional and therefore, not all qualified GPs have been exposed to obstetrics and gynaecology. Several participants suggested that incorporating miscarriage communication training into emergency department rotations could ensure that all doctors, regardless of their specialty, are provided with the skills to handle miscarriage consultations with sensitivity and empathy.

### Strengths and weaknesses

Although the sample size was small, a key strength of this study is its dual focus on both consumers and GPiTs, offering a comprehensive understanding of the challenges in miscarriage care from both perspectives. The diverse participant demographics, including GPiTs and consumers from different states of Australia, enhanced the breadth of the findings. Thematic analysis allowed for a deep exploration of emotional and professional dynamics, while the inclusion of GPiTs with personal experience of miscarriage added valuable information to the study. As a qualitative study, the results were not intended to be generalisable to a wider population; however, in noting this, the findings with consumers and GPiTs align closely with previous research in the field. One limitation was the largely homogenous consumer group, as majority of consumers were caucasian Australian women aged 20–40. As such, there was limited information regarding the specific challenges experienced by women from different cultural backgrounds. The reliance on self-reported data may have introduced potential recall bias, and the focus group format may have led to participant selection bias affecting the accuracy and diversity of responses.

## Conclusion

The findings from this study have several important implications for clinical practice. Firstly, the delivery of emotional care in miscarriage consultations must be incorporated into routine care. This includes not only acknowledging the loss but also addressing feelings of guilt, providing emotional reassurance, and offering follow-up support. Furthermore, all GPiTs should be trained in delivering clear, concise and empathetic information, which should be tailored to each patient’s needs. This includes discussing the physical and emotional aspects of miscarriage in a way that is both realistic and sensitive. Finally, incorporating miscarriage counselling training into medical education is essential for improving the quality of care delivered by healthcare providers and ensures that women receive the support they need during what is often a challenging experience.

## Data Availability

The datasets generated and/or analysed during the current study are not publicly available due to the confidential nature of the discussions taken place but are available from the corresponding author on reasonable request.

## Abbreviations

HCPs: health care professionals
GP: General Practitioners
GPiTs: GPs–in–training
D + C: Dilatation and curettage
RACGP: Royal Australian College of General Practitioners
DRANZCOG: Diploma of the Royal Australian and New Zealand College of Obstetricians and Gynaecologists
ED: Emergency department
